# Distribution of Exostosis on the Teeth

**Published:** 1897-04

**Authors:** H. W. C. Bodecker


### Distribution of Exostosis on the Teeth.

H. W. C. BODECKER, B. S.

Exostosis may be found at any point on the roots of the teeth. An examination of about fifteen hundred teeth showed that exostosis is most frequently met with upon the upper molars. In Table No. 1 I have tried to show the distribution of exostosis in the arch; while in Table No. 2 the frequency of its occurrence on the individual teeth has been tabulated.

TABLE NO. 1.

| | EXAMINED. | EXOSTOSED. | PERCENT. |
|---|---|---|---|
| Incisors | 62 | 4 | 6 |
| Cuspids | 100 | 5 | 5 |
| Bicuspids | 138 | 12 | 8.6 |
| Lower molars | 260 | 31 | 12 |
| Upper molars | 340 | 90 | 26.5 |
| Total | 900 | 142 | 15.7 |

Another series of teeth were examined, with the following results:

| | EXAMINED. | EXOSTOSED. | PERCENT. |
|---|---|---|---|
| Incisors | 66 | 4 | 6 |
| Upper centrals | (32) | 3 | |
| Upper laterals | (33) | 1 | |
| Lower centrals | (15) | 0 | |
| Lower laterals | (15) | 0 | |
| Cuspids | 96 | 5 | 5.2 |
| Upper | (50) | 3 | |
| Lower | (46) | 2 | |
| Bicuspids, upper | 65 | 7 | 9.1 |
| First bicuspid | (32) | 4 | |
| Second bicuspid | (33) | 3 | |
| Bicuspids, lower | 60 | 5 | 8.3 |
| First bicuspid | (30) | 3 | |

| | | | |
|---|---|---|---|
| Second bicuspid | (30) | 2 | |
| Molars, upper | 175 | 40 | 23 |
| First molar | (55) | 11 | |
| Second molar | (60) | 15 | |
| Third molar | (60) | 14 | |
| Molars, lower | 165 | 18 | 10.9 |
| First molar | (50) | 6 | |
| Second molar | (50) | 9 | |
| Third molar | (65) | 3 | |
| Total | 627 | 79 | 12.4 |

These figures can not, however, be accepted as pertaining to persons of all ages, as the teeth examined were such as are obtained from extracting offices, and among these, teeth of older people always predominate.

Of the two varieties, the diffused is met with more frequently. Among 84 exostosed teeth, 61 (72 percent) were found to be of the diffused variety, while but 23 (28 percent) were cases of circumscribed exostosis. The circumscribed variety is found mostly at the apex, though it may occur on the body of the root. The diffused variety may appear with equal frequency on any part of the root.

Occasionally only one root is affected, or one of the roots may be deformed by circumscribed exostosis, while on the others we find the diffused variety.

The size of the osteoma varies greatly. It may appear only as a slight elevation ; or it may, as in some cases, grow to be equal in volume to the tooth itself.

In form the circumscribed variety is always more or less globular, while the diffused may be only a thin layer or an outgrowth uniting the several roots ; or it may even develop so far as to form a cup-like growth extending beyond the apex of the root.

In every one of nine cases of union between two adjacent teeth, the tissue binding them together was exostoma of the diffused variety. Exostosis, therefore, may occur in a greater or lesser degree upon any portion of a root or upon any tooth, though the upper teeth, and especially the upper molars, are most likely to be affected by this disease.

